# Heat Stroke in a Young Athlete With Attention Deficit Hyperactivity Disorder on Stimulant Medication

**DOI:** 10.7759/cureus.68020

**Published:** 2024-08-28

**Authors:** Ozair Qazi, Ahmad Mohammed, Samrawit W Zinabu, Sair Ahmad Tabraiz, Haris Ansari, Patrice Lexima, Tatiana Balabanis, Aaron Mack, Rediet Tefera Atalay, Miriam B Michael

**Affiliations:** 1 Psychiatry, Howard University Hospital, Washington, DC, USA; 2 Orthopedics, Howard University Hospital, Washington, DC, USA; 3 Internal Medicine, Howard University Hospital, Washington, DC, USA; 4 Internal medicine, Punjab Medical College, Allied Hospital Faisalabad, Faisalabad, PAK; 5 Pediatric Medicine, Howard University Hospital, Washington, DC, USA; 6 Internal Medicine, University of Maryland, Baltimore, USA

**Keywords:** amphetamine, global warming and rising temperature, heat stroke, heat-related injury, attention-deficit/hyperactivity disorder (adhd)

## Abstract

Attention-deficit/hyperactivity disorder (ADHD) is a neurodevelopmental disorder characterized by persistent patterns of inattention, hyperactivity, and impulsivity that significantly impair daily functioning and quality of life. Although often diagnosed in childhood, ADHD symptoms frequently persist into adolescence and adulthood. Heat stroke, a severe medical condition characterized by central nervous system dysfunction, seizures, and extreme hyperthermia, can result in mortality even with medical intervention. Notably, exertional heat stroke remains a leading cause of sudden death among young athletes and individuals engaged in strenuous physical activity. We present a case of a young athlete diagnosed with ADHD and prescribed amphetamine and dextroamphetamine (Adderall), who presented with heat stroke, partly due to his medication.

## Introduction

Attention-deficit/hyperactivity disorder (ADHD) affects 5%-7.2% of youth and 2.5%-6.7% of adults globally, with a notably higher prevalence in the United States, particularly among children, where the rate is higher than 9% [1]. This disorder significantly impacts various aspects of individuals' lives, including relationships, emotional health, academic performance, and daily functioning [2]. Despite extensive research over the past decade, diagnosing and treating ADHD remains challenging [3]. Early detection, accurate diagnosis, and appropriate intervention are essential to mitigating the long-term effects associated with ADHD [3].

Medications, especially stimulants like amphetamines and methylphenidate, are crucial in managing symptoms such as hyperactivity, inattention, and impulsivity in individuals with ADHD [4]. Understanding the link between ADHD medications and heat-related injuries is critical, particularly as sports activities escalate in intensity in the context of rising global temperatures [5]. Stimulant medications, including methylphenidate and amphetamines, interfere with the body’s ability to regulate heat, increasing the risk of heat-related illnesses [6]. This risk is especially significant for young athletes with ADHD, who may experience heightened heat stress during intense physical activities or exertion [7,8]. Physicians need to be aware of these factors to ensure the safety of young athletes with ADHD who are being treated with stimulant medications.

## Case presentation

A 17-year-old African American male with a history of ADHD, recently initiated on a twice-daily regimen of 20 mg amphetamine and dextroamphetamine, presented to the ED with syncope. The patient had been participating in a summer tennis program and experienced the syncopal episode during drills on asphalt courts following a two-mile warm-up run. The ambient temperature was documented as 98 degrees Fahrenheit during the incident. The patient reported several prior near-syncopal events occurring around mid-day when temperatures were at their highest, noting the need to cool down by throwing water over his head and face and running cold water on his arms and legs.

Upon initial assessment, the patient was alert and oriented, exhibiting a blood pressure of 95/62 mmHg, a temperature of 103.2 degrees Fahrenheit, a respiration rate of 16 breaths per minute, and a pulse of 110 beats per minute. Immediate interventions included the administration of one liter of Ringer's lactate intravenously and the application of ice packs. Upon presentation to the hospital, the patient's vital signs had improved to a temperature of 98.7 degrees Fahrenheit, a blood pressure of 110/72 mmHg, and a pulse of 90 beats per minute. The physical examination revealed a well-developed, well-nourished individual with clear lungs, a soft abdomen, and extremities without bruising, rash, or deformity. Laboratory analysis indicated a blood urea nitrogen (BUN) of 45 mg/dL, creatinine of 1.2 mg/dL, and creatine phosphokinase (CPK) of 1403 U/L. The patient was monitored in the ED for several hours, receiving a second liter of fluids. Repeat laboratory tests 6 hours later showed a BUN of 12 mg/dL, creatinine of 0.99 mg/dL, sodium of 140 mmol/L, and potassium of 3.5 mmol/L. The patient was subsequently discharged. The initial and repeat laboratory results are shown in Table 1.

**Table 1:** Initial and repeat laboratory test results for the patient.

Test	Initial Result	Repeat Result (6 hours later)
Blood urea nitrogen (BUN)	45 mg/dL	12 mg/dL
Creatinine	1.2 mg/dL	0.99 mg/dL
Creatine phosphokinase (CPK)	1403 U/L	-
Sodium	-	140 mmol/L
Potassium	-	3.5 mmol/L

## Discussion

**Figure 1:**
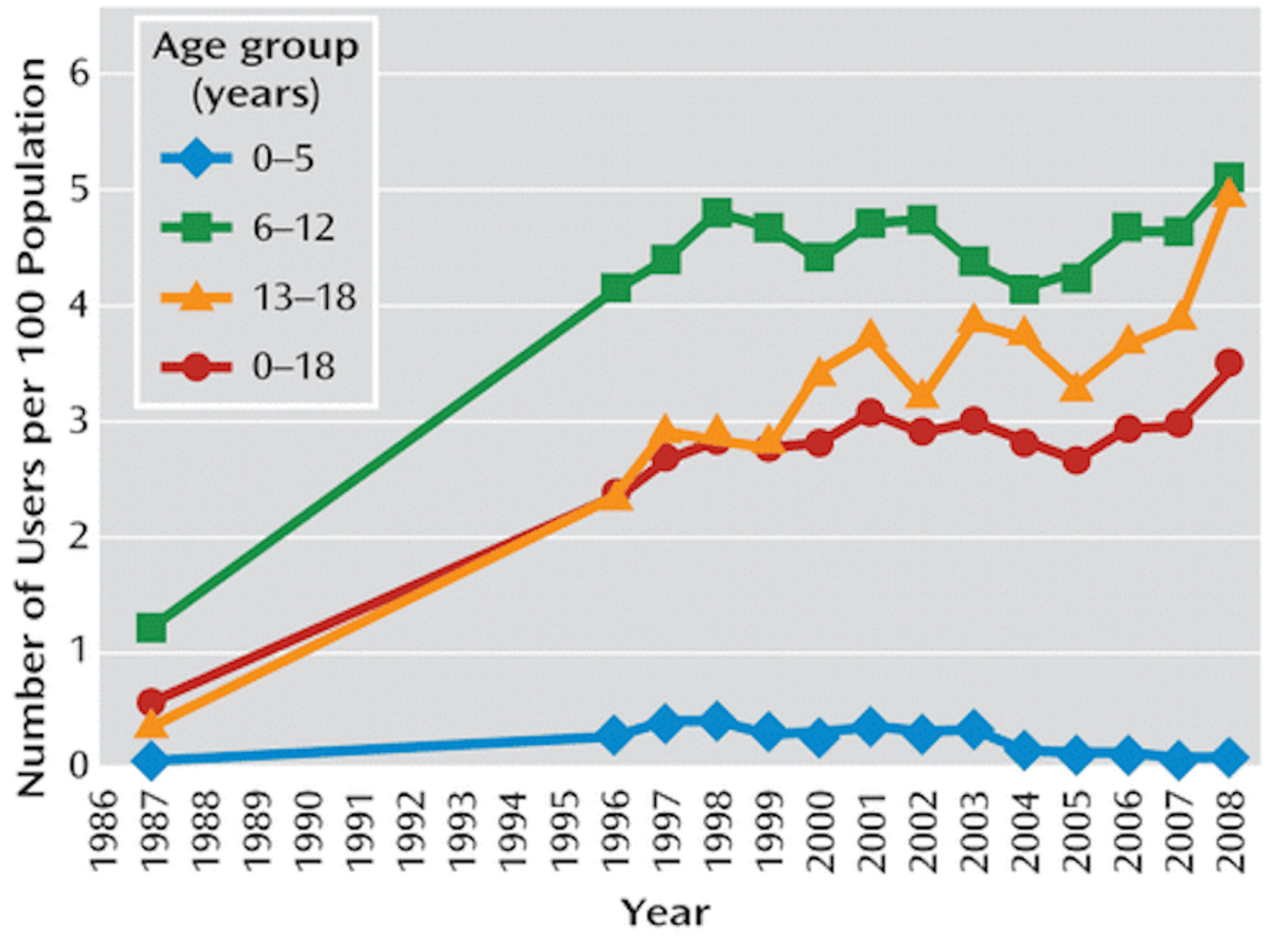
Trends in prevalence of stimulant use in the U.S. population aged 18 and younger, 1987–2008. Figure taken from Zuvekas SH and Vitiello B [11]. This article is licensed under a Creative Commons Attribution 4.0 International License, which permits use, sharing, adaptation, distribution, and reproduction in any medium or format, as long as appropriate credit is given to the original author(s) and the source. For more details, visit http://creativecommons.org/licenses/by/4.0/.

According to the U.S. National Statistics report from 2016-2019, approximately 6 million children aged 3-17 were diagnosed with ADHD, constituting 9.8% of this age group. The incidence was higher in boys (13%) compared to girls (6%), with a higher prevalence of ADHD among Black and White children compared to Asian or Hispanic children [9]. Additionally, data from the 2016 National Survey of Children's Health indicate that approximately 62% of children aged 2-17 with recurrent ADHD were treated with medication [10]. Data from the American Journal of Psychiatry (Figure 1) reveal that the use of stimulants has seen a significant upward trend since 1987 across all age groups, except in children five years old or younger.

**Figure 2:**
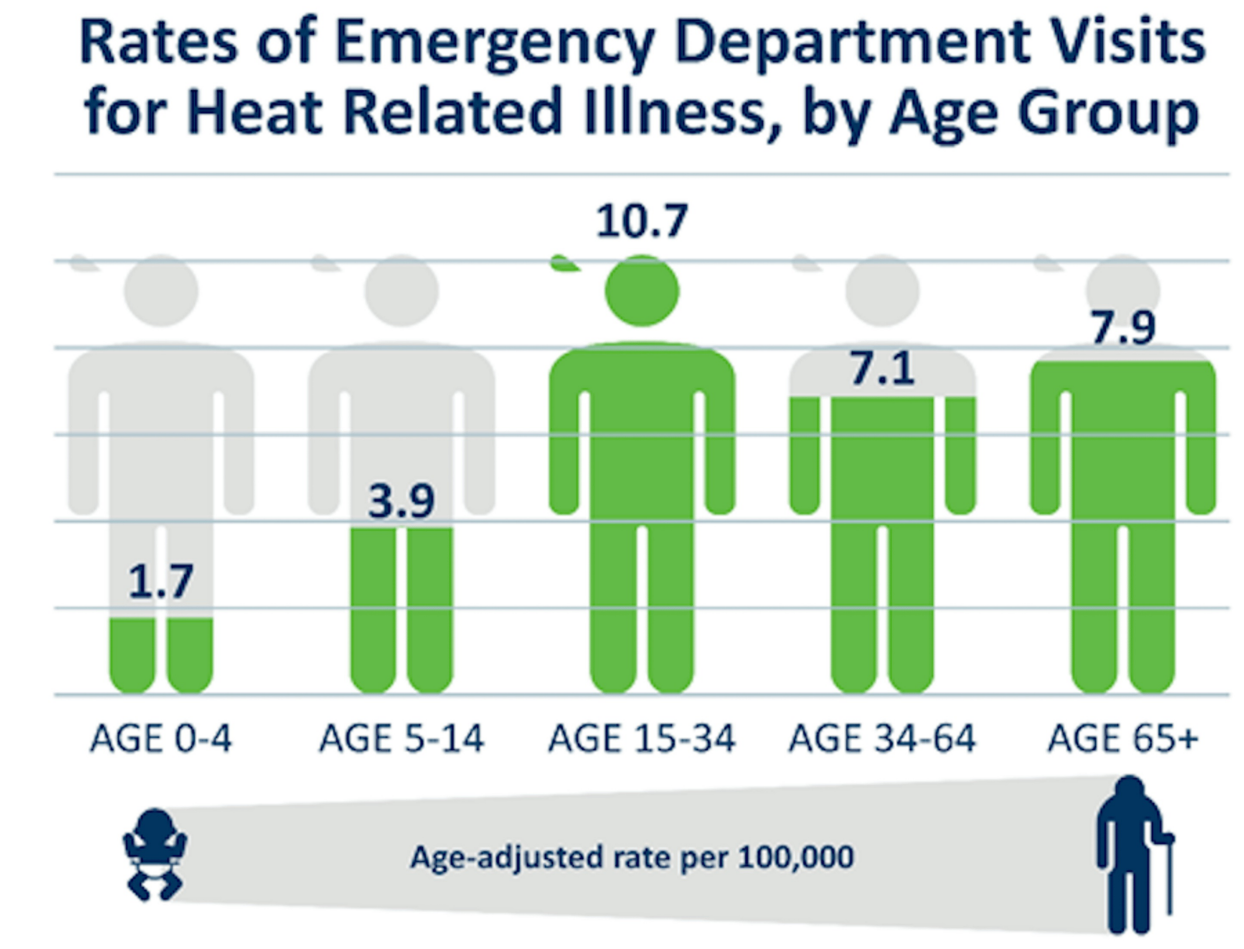
Rates of emergency department visits for heat-related illness by age group. Figure taken from the Minnesota Department of Health [13] and used with permission.

Stimulant medications, such as amphetamines and methylphenidate, exert their therapeutic effects by modulating neurotransmitters like dopamine and norepinephrine, which are intrinsically linked to motivation, reward, and attention processes [1]. However, these pharmacological actions can also disrupt thermoregulation by elevating metabolic rate and heat production, thereby increasing core body temperature. Moreover, stimulants impair heat dissipation by altering normal sweating patterns and thermoregulatory responses [12]. This combined effect significantly increases the risk of dehydration, hyperthermia, and other heat-related complications, particularly during strenuous physical activity or exposure to high ambient temperatures. This is likely why the highest incidence of heat-related illness is observed in the 15-34 age range, a demographic often engaged in high-intensity physical activities and sports (Figure 2). Their physiological response to heat, coupled with these activities, makes them particularly susceptible to heat-related illnesses. Their elevated risk underscores the importance of preventive measures and education about the dangers of heat exposure in this age group.

The impact of stimulants on the sympathetic nervous system further exacerbates the risk of heat-related illnesses. By stimulating this system, these medications elevate heart rate, blood pressure, and respiration, contributing to an overall increase in body temperature. Furthermore, stimulants have been shown to alter metabolic processes, accelerating calorie expenditure and suppressing appetite [14]. Consequently, athletes with ADHD who are treated with stimulants face a heightened risk of heat-related illnesses due to these multifaceted effects on the body's thermoregulatory system, especially during intense physical exertion [8].

The rising global temperatures due to climate change are increasingly endangering public health, particularly through heat-related illnesses. Athletes participating in outdoor sports are especially vulnerable to these environmental conditions. Exertional heat illness, encompassing a spectrum of conditions ranging from mild heat syncope to life-threatening heat stroke, has been the research focus of Pryor JL et al., highlighting the urgent need for preventive strategies involving sports coaches, parents, and policymakers [15].

Preventing heat stroke in athletes necessitates aggressive and immediate intervention, including early recognition of symptoms, rapid cooling techniques, and appropriate medical management [16]. Current research underscores the importance of proactive measures by healthcare providers, parents, and coaches to mitigate the risks associated with stimulant use in athletes. These measures may include adjusting activity levels based on environmental conditions, ensuring adequate hydration, and monitoring athletes for signs of heat-related distress [7].

Athletes with ADHD who use stimulant medications warrant heightened vigilance and specialized care due to their increased susceptibility to heat-related illnesses. Healthcare providers responsible for the well-being of these athletes should possess comprehensive knowledge of ADHD diagnosis and treatment modalities, including stimulant and non-stimulant options. A thorough understanding of how stimulant medications can influence athletic performance, both positively and negatively, is paramount. Additionally, healthcare providers must be well-versed in the potential side effects of these medications, particularly those related to thermoregulation and cardiovascular function. By prioritizing education, raising awareness, and adhering to established guidelines and best practices, healthcare providers can effectively minimize the risks associated with stimulant medication use in athletes with ADHD [17].

## Conclusions

This case study underscores the complex interplay between the use of stimulant medication for ADHD in athletes and their heightened vulnerability to heat-related illnesses. The findings highlight the urgent need for improved heat mitigation strategies and surveillance, particularly in the context of rising global temperatures. With ADHD being a prevalent diagnosis and stimulant medications commonly prescribed for its treatment, there is a growing population of young athletes potentially at risk. Heightened awareness of the effects of these medications on athletes exposed to elevated temperatures is crucial, and further research is warranted to investigate the specific side effects and develop tailored interventions. Heat-related illness is a serious concern, and athletes taking ADHD medications represent a particularly vulnerable demographic. Proactive measures to mitigate these risks are essential to ensure the safety and well-being of this population.

## References

[1] Abdelnour E, Jansen MO, Gold JA (2022). ADHD diagnostic trends: increased recognition or overdiagnosis?. Mo Med.

[2] Schneider BN, Enenbach M (2014). Managing the risks of ADHD treatments. Curr Psychiatry Rep.

[3] Drechsler R, Brem S, Brandeis D, Grünblatt E, Berger G, Walitza S (2020). ADHD: current concepts and treatments in children and adolescents. Neuropediatrics.

[4] Faraone SV (2018). The pharmacology of amphetamine and methylphenidate: relevance to the neurobiology of attention-deficit/hyperactivity disorder and other psychiatric comorbidities. Neurosci Biobehav Rev.

[5] Matuszak JM (2020). Attention deficit/hyperactivity disorder (ADHD). Mental Health in the Athlete.

[6] Thoenes MM (2011). Heat-related illness risk with methylphenidate use. J Pediatr Health Care.

[7] Parr JW (2011). Attention-deficit hyperactivity disorder and the athlete: new advances and understanding. Clin Sports Med.

[8] Stewman CG, Liebman C, Fink L, Sandella B (2018). Attention deficit hyperactivity disorder: unique considerations in athletes. Sports Health.

[9] (2022). Data and Statistics on ADHD. https://www.cdc.gov/adhd/data/?CDC_AAref_Val=https://www.cdc.gov/ncbddd/adhd/data.html.

[10] Danielson ML, Bitsko RH, Ghandour RM, Holbrook JR, Kogan MD, Blumberg SJ (2018). Prevalence of parent-reported ADHD diagnosis and associated treatment among U.S. children and adolescents, 2016. J Clin Child Adolesc Psychol.

[11] Zuvekas SH, Vitiello B (2012). Stimulant medication use in children: a 12-year perspective. Am J Psychiatry.

[12] Judge BS, Rusyniak DE (2009). Clinical Neurotoxicology: Syndromes, Substances, Environments. Illicit Drugs I: Amphetamines.

[13] Heat and Vulnerability in Minnesota and Wisconsin. (2022) (2022). Heat and Vulnerability in Minnesota and Wisconsin. https://www.health.state.mn.us/communities/environment/tracking/projects/heatvulnerabilitymnwisc.html .

[14] Watson S (2017, August 25) (2017). How ADHD medication can affect your weight. https://www.webmd.com/add-adhd/medication-weight.

[15] Pryor JL, Périard JD, Pryor RR (2020). Predisposing factors for exertional heat illness. Exertional Heat Illness.

[16] Howe AS, Boden BP (2007). Heat-related illness in athletes. Am J Sports Med.

[17] Kreher JB (2012). Attention deficit/hyperactivity disorder (ADHD) in athletes. Int J Athl Ther Train.

